# Machine learning-based approaches for cancer prediction using microbiome data

**DOI:** 10.1038/s41598-023-38670-0

**Published:** 2023-07-21

**Authors:** Pedro Freitas, Francisco Silva, Joana Vale Sousa, Rui M. Ferreira, Céu Figueiredo, Tania Pereira, Hélder P. Oliveira

**Affiliations:** 1 INESC TEC - Institute for Systems and Computer Engineering, Technology and Science, 4200-465 Porto, Portugal; 2 FEUP - Faculty of Engineering, University of Porto, 4200-465 Porto, Portugal; 3 FCUP -Faculty of Science, University of Porto, 4150-177 Porto, Portugal; 4 Ipatimup - Institute of Molecular Pathology and Immunology of the University of Porto, 4200-135 Porto, Portugal; 5 i3S - Instituto de Investigação e Inovação em Saúde, University of Porto, 4200-135 Porto, Portugal; 6 FMUP - Faculty of Medicine, University of Porto, 4200-319 Porto, Portugal

**Keywords:** Microbiome, Biomedical engineering, Predictive markers, Cancer, Computer science

## Abstract

Emerging evidence of the relationship between the microbiome composition and the development of numerous diseases, including cancer, has led to an increasing interest in the study of the human microbiome. Technological breakthroughs regarding DNA sequencing methods propelled microbiome studies with a large number of samples, which called for the necessity of more sophisticated data-analytical tools to analyze this complex relationship. The aim of this work was to develop a machine learning-based approach to distinguish the type of cancer based on the analysis of the tissue-specific microbial information, assessing the human microbiome as valuable predictive information for cancer identification. For this purpose, Random Forest algorithms were trained for the classification of five types of cancer—head and neck, esophageal, stomach, colon, and rectum cancers—with samples provided by The Cancer Microbiome Atlas database. One versus all and multi-class classification studies were conducted to evaluate the discriminative capability of the microbial data across increasing levels of cancer site specificity, with results showing a progressive rise in difficulty for accurate sample classification. Random Forest models achieved promising performances when predicting head and neck, stomach, and colon cancer cases, with the latter returning accuracy scores above 90% across the different studies conducted. However, there was also an increased difficulty when discriminating esophageal and rectum cancers, failing to differentiate with adequate results rectum from colon cancer cases, and esophageal from head and neck and stomach cancers. These results point to the fact that anatomically adjacent cancers can be more complex to identify due to microbial similarities. Despite the limitations, microbiome data analysis using machine learning may advance novel strategies to improve cancer detection and prevention, and decrease disease burden.

## Introduction

Cancer is one of the leading causes of death worldwide, accounting for almost 10 million deaths in 2020^1^. In the same year, over 19 million new cases of cancer were diagnosed. The global cancer burden can be reduced by implementing strategies for prevention complemented with early detection and efficient treatment approaches. Cancer comprises a heterogeneous group of diseases that result from the interaction between individual genetic susceptibility factors and environmental carcinogens of physical, chemical, and infectious nature^2^.

The human microbiome represents the entire microbial population that colonizes the human body, including bacteria, viruses, and fungi^3^. Perturbations to the microbiome composition of an individual, also known as dysbiosis, have been associated with numerous diseases, including cancer^4–7^. The microbiome may influence cancer development by these microbial community changes, by a direct effect of specific microbial species, or by indirect modulation of host cells through the secretion of toxin or metabolites^8,9^. Through comprehensive analysis of the microbiome of tumors and adjacent normal tissues across a diverse range of human cancers, recent studies have revealed the presence of microbes within tumors, establishing that various tumor types exhibit distinct microbial signatures^10–13^. Such knowledge is crucial not only for understanding the relationship between the microbiome, cancer cells, and the tumor microenvironment, but also for the design of novel approaches for cancer detection and prevention.

The advances in high-throughput methods, including next-generation sequencing (NGS), allowed the progress in both cancer and microbiome research by providing unprecedented sensitivity. However, this increased sensitivity poses a challenge as it can also detect contaminant DNA, leading to potential misinterpretation of microbiome data. Contaminant DNA is frequently present in commonly used DNA extraction kits and laboratory reagents, and it significantly influences the analysis outcomes of low microbial biomass samples^14,15^. Consequently, there is an urgent need to implement robust controls in microbiome research to enhance the reliability and integrity of microbiome studies. Furthermore, high-throughput methods generate substantial amounts of data, which require powerful and improved computational tools for accurate analysis. In this sense, the use of artificial intelligence (AI), and more precisely, the application of machine learning (ML) algorithms, represents a major approach to data analysis and predictive modeling to explore the cancer-associated microbiome. Recent examples have applied these types of algorithms in classification problems. A Logistic Regression algorithm was implemented as a predictive model for disease classification based on microbial information extracted from 806 shotgun metagenomic samples of Chinese patients (170 with type 2 diabetes, 130 with rheumatoid arthritis, 123 with liver cirrhosis, and 383 controls)^16^. From the identified microbiome of the study population, 300 biomarkers were selected, which allowed for the model to achieve an F1 score of 91.42% and an average AUC of 94.75%, showcasing the importance of ML as an approach to microbial feature selection and predictive modeling of disease. A Random Forest (RF) model was implemented for the classification of colorectal cancer based on microbial information of stool samples from 490 patients obtained by 16S rRNA gene sequencing^17^. Reporting an AUC of 84.7%, this approach revealed results that exceeded existing screening methods, showing the potential of microbiome-based predictive modeling as a complementary technique to common practices. Another study used a Support-vector Machine (SVM) algorithm to determine the optimal gut microbial profile for non-invasive lung cancer diagnosis^7^. By profiling the gut microbiota composition of two independent cohorts through 16S rRNA gene sequencing, the SVM model achieved an AUC of 97.6% in a discovery cohort of 42 patients and 65 controls, and an AUC of 76.4% in a validation cohort of 34 patients and 40 controls. Gradient Boosting (GB) models have also been trained to discriminate between and within types and stages of cancer-based on the microbiome^18^. Microbial reads were extracted from whole-genome and whole-transcriptome sequencing studies provided by The Cancer Genome Atlas (TCGA) for 33 types of cancer. In a one versus all classification approach, the GB model produced an AUC of 99% when discriminating cancers such as esophageal, colon, and rectum cancers, showing that each cancer has a unique microbial profile that successfully distinguishes cancer types. In the field of deep learning (DL), a deep neural network (DNN) approach was implemented to evaluate the potential of the microbiome as a non-invasive biomarker to assess the risk of colorectal cancer^19^. The gut microbiota of 183 home collected stool samples was profiled through 16S rRNA analysis and the neural network achieved an AUC of 87% when discriminating for colorectal polyps.

The previous studies highlight ML as a suitable approach for the analysis of the microbiome-cancer relationship, with a wide range of possible applications, although highly dependent on the data available. Moreover, most of these studies focused on the assessment of a microbial profile to discriminate a specific cancer type. Nevertheless, it remains to be determined whether a single microbial profile can be suitable to classify various types of cancer. In this work, we used cancer microbial data from The Cancer Microbiome Atlas (TCMA)^20^, which is a publicly available database comprising curated and decontaminated tissue microbial profiles of patients from five TCGA projects, namely head and neck squamous cell carcinoma (HNSC), esophageal carcinoma (ESCA), stomach adenocarcinoma (STAD), colon adenocarcinoma (COAD), and rectum adenocarcinoma (READ). Using these data, the main goal of this study was to develop a supervised ML model that can distinguish cancer types based on the analysis of their specific microbial information, exploring the microbiome as valuable predictive information for cancer identification. For this purpose, one versus all and multi-class classification studies were conducted in ascending order in terms of cancer site specificity, which allow to assess the development of the microbial data as an effective predictor for the different types of cancers.

## Material and methods

### Data description

The data was acquired through TCMA^20^ at https://tcma.pratt.duke.edu. This database ensures, on the correspondent published description article, that the necessary ethical approvals regarding data access were obtained. It provides a total sample size of 620 samples and has the possibility to choose the taxonomic level of the microbial information from *phylum*, *class*, *order*, *family*, and *genus*. In this work, the microbial data was obtained at the *genus* level, which comprised 221 different *genera* available. The TCMA database provides its information in two different but complementary datasets - the microbial dataset (unique sample and the relative abundance of the respective *genus* in a given sample) and the metadata dataset (unique sample and information on sample type, cancer type, and corresponding TCGA project).

### Data pre-processing

The first step for data pre-processing consisted of an overall assessment of the datasets in order to discard any information that was either incomplete or not relevant to this investigation. Regarding sample type, this attribute can represent the tumor tissue of the primary tumor (PT) or the normal tissue adjacent to the tumor normal (STN). Of the 620 samples, 512 (82.58%) were PT samples. Since the premise of this study was to distinguish cancer types based on the analysis of their specific microbial information, the remaining 108 STN samples were discarded as they fall outside the scope of this study. From the cancer type and corresponding TCGA project, the sample distribution for the various cancers was as follows: HNSC (155 samples), STAD (127 samples), COAD (125 samples), ESCA (60 samples), and READ (45 samples).

Furthermore, it was observed that among the 221 genera initially present in the dataset, certain genera were not found in any of the samples. These non-present genera were excluded from the analysis as they did not contribute any informative data to the study. Consequently, 90 genera were removed from the initial set of 221, resulting in a final dataset of 512 samples with the relative abundance of 131 genera used as features for the ML models (refer to Supplementary Table S1). Notably, the data from the TCMA database was already normalized, considering the proportion of each genus in relation to the total amount of bacteria present in the sample, eliminating the need for further normalization steps.

### Experiment design and metrics

Given that TCMA contains an extremely sparse microbial dataset, it becomes paramount to provide the ML models with the most informative features possible, through methods such as feature selection. In this sense, the ML approach established was based on the implementation of the RF, an algorithm with an intrinsic feature selection mechanism. Code was developed through the Python programming language using the scikit-learn package^21^. The RF models were trained and tested on separate, stratified sampling splits of 85% and 15% of the dataset, respectively, with the same splits being used to ensure comparability between experiments. Hyper-parameter tuning was conducted by performing grid search optimization with stratified 5-fold cross-validation on the training split, aiming to maximize the balanced accuracy of the RF model on the validation set.

**Figure 1:**
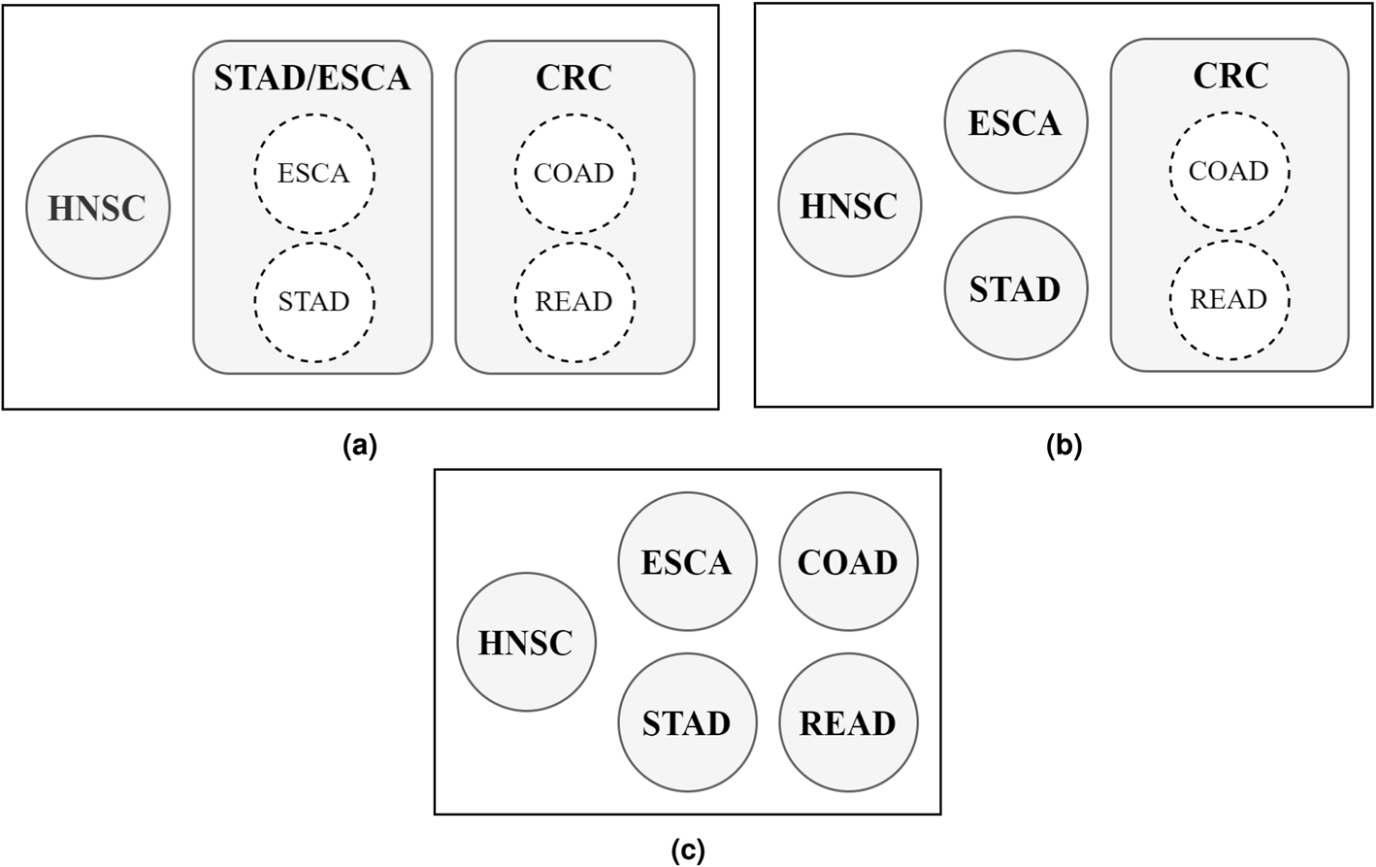
Multi-class classification studies conducted with cancer types grouped according to (**a**) 3-class, (**b**) 4-class, and (**c**) 5-class approaches.

A total of 4 granularity levels of analysis were conducted to assess the predictive power of the microbial data on distinct levels of specificity based on the anatomic location of the different types of cancer. In the initial study, a one-*vs*-all approach was implemented to the classification problem, allowing to gain a first insight into the performance of the RF model when discriminating each cancer specifically. Then, a second study was carried out by aggregating the five cancer types given by the TCMA database—HNSC, STAD, COAD, ESCA, and READ—into 3 major classes based on anatomical proximity: HNSC, STAD/ESCA, and colorectal cancer (CRC)^17,22,23^ (Fig. 1a). This analysis served as a starting point to evaluate the ability of the microbial data in the classification of distinct anatomical areas where the cancer was located, and would further allow the investigation to progress in a direction of higher specificity in terms of the cancer site. In the third study, an even more fine-grained approach was implemented, STAD and ESCA were separated into the original classes while maintaining CRC as a combination of COAD and READ (Fig. 1b). In the fourth and final study, which is the most fine-grained approach, CRC was then split into COAD and READ, thus resulting in the five initial classes provided by TCMA (Fig. 1c).

**Figure 2:**
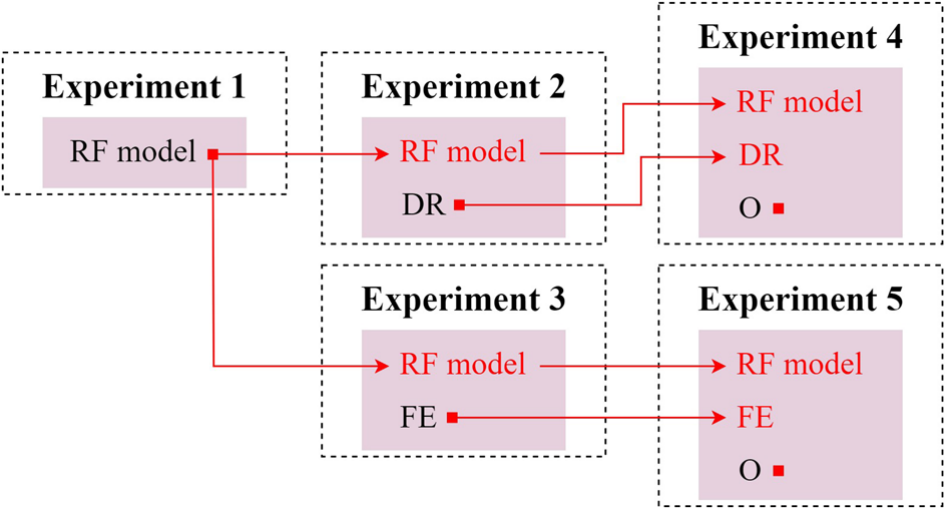
Experiment pipeline for the learning models development, with the isolated implementation of the RF algorithm (Experiment 1), the implementation of dimensionality reduction (DR) and feature engineering (FE) alongside the tuned RF (Experiments 2 and 3, respectively), and the final application of oversampling (O) with the previous techniques (Experiments 4 and 5). Red squares indicate that hyper-parameter tuning is being performed to a specific technique while red arrows indicate the use of a technique tuned in a former experiment.

For each study of the granularity of the detection, the experiment pipeline for the learning model development is illustrated in Fig. 2 and consists of the following experiments:

- *Experiment 1:* initial isolated implementation of the algorithm and the assessment of its performance after hyper-parameter tuning was performed;
- *Experiment 2:* in an attempt to provide the models with a simpler and more separable feature space and ultimately increase the performance of the baseline RF models, several dimensionality reduction techniques were tested, including: Sparse Principal Component Analysis (SPCA), Non-negative Matrix Factorization (NMF), and Linear Discriminant Analysis (LDA). These techniques were applied independently alongside the tuned RF algorithm, with hyper-parameter tuning being performed for the auxiliary techniques;
- *Experiment 3:* in the cases where the application of dimensionality reduction to the dataset failed to meaningfully improve the performance of the baseline RF model, a further experiment was developed based on a feature engineering approach where the components given by the dimensionality reduction techniques were added to the dataset while maintaining the original feature set;
- *Experiment 4:* taking into consideration the differences in sample size for each class, measures to counter the possible negative effects of existing class imbalance must be adopted. In this sense, with the main objective of improving the performance of RF models when classifying samples from the minority classes, data augmentation was also applied, with Random Oversampling and SVM-SMOTE being used as oversampling techniques. In this context, each model was implemented with dimensionality reduction and oversampling, simultaneously. The dimensionality reduction technique that resulted in the most improvement of performance in Experiment 2 was first applied to the dataset, followed by oversampling techniques that were tuned in this context;
- *Experiment 5:* similar to experiment 4 but with the implementation of feature engineering instead of dimensionality reduction.

Finally, in regard to the performance evaluation metrics for the ML model, it is important to consider the different sample sizes that exist across classes. Therefore, balanced accuracy was chosen as the metric to assess the performance of the ML models, since it takes into consideration these dissimilarities and does not indicate high performances when the model takes advantage of the majority classes. Furthermore, confusion matrices were also constructed since they allow to better understand of the behavior of the model when performing sample classification for each specific class.

#### Experiment 1: baseline

The first experiment consisted of an isolated implementation and hyper-parameter tuning of the RF algorithm which would serve as a baseline model for the next experiments regarding dimensionality reduction/feature engineering and oversampling. Hyper-parameters and their value range used for tuning are found in Supplementary Table S2.

#### Experiment 2 and 3: dimensionality reduction/feature engineering

The tuning of the dimensionality reduction techniques was conducted with hyper-parameters of the predictive model fixed according to the baseline. Besides LDA, the number of components given by SPCA and NMF ranged from 10 to 100 in the dimensionality reduction approach, and from 4 to 64 in the feature engineering approach. In the case of LDA, as its output is required to have a dimension inferior to a given number of classes *n*, the number of components ranged between 1 and $\textit{n}-1$ for both approaches.

#### Experiment 4 and 5: dimensionality reduction/feature engineering and oversampling

Following the implementation of dimensionality reduction/feature engineering in the dataset, oversampling was applied in the training split, aiming to improve the performance of the models mainly when classifying samples belonging to the minority classes. Classes were oversampled based on three distinct approaches depending on the magnitude of oversampling. In the first approach, each class was oversampled until it reached an equal number of samples as the majority class, which would remain with the original sample size. In the second and third approaches, the majority class was oversampled by 50% and 100%, respectively, with all other classes matching its sample size.

## Results and discussion

### Study 1: one-versus-All

In the one-versus-all study, a total of 5 independent analyses were conducted. For each analysis, a different cancer class was targeted (positive class) and the samples from the remaining classes were grouped together into one major class (negative class), resulting in a binary classification problem.

**Figure 3:**
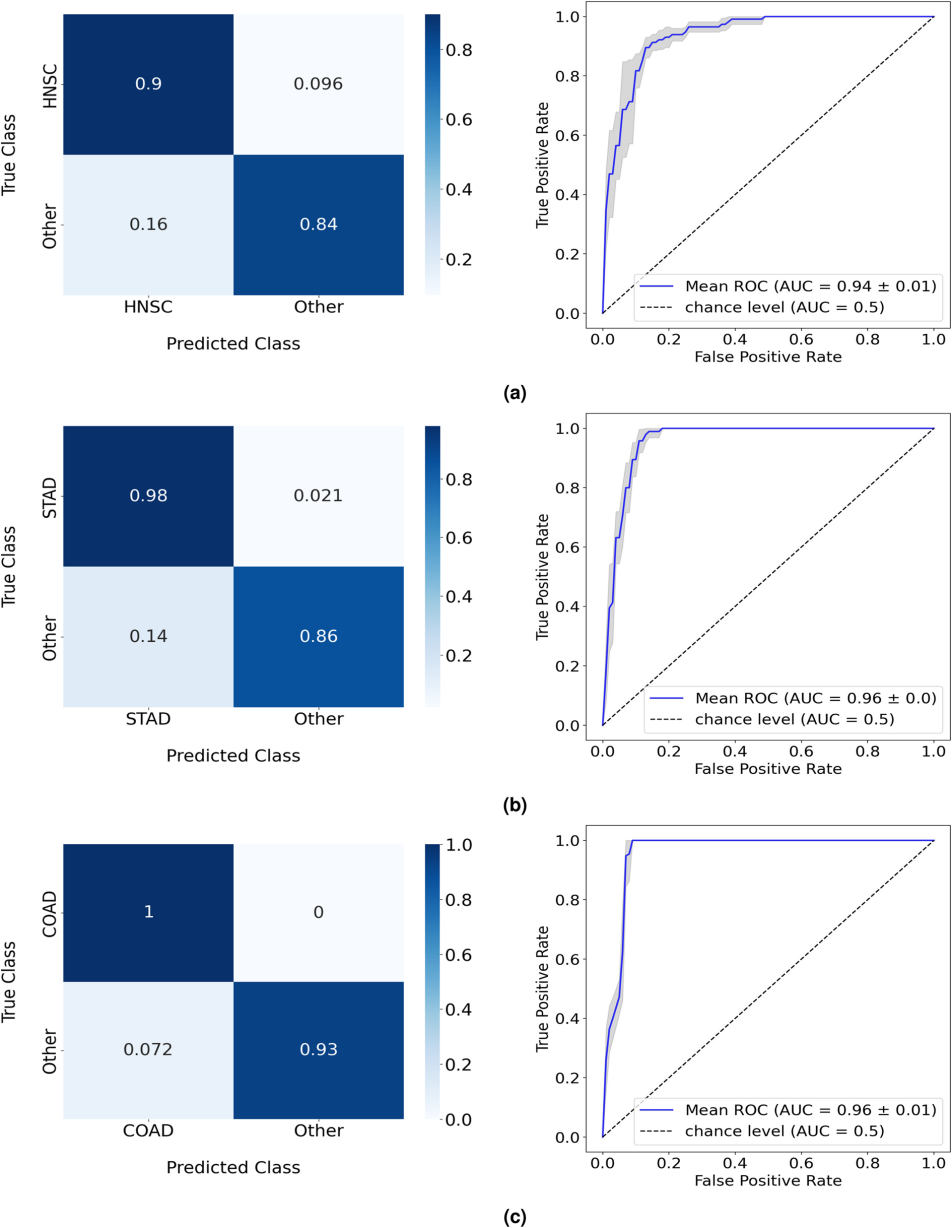

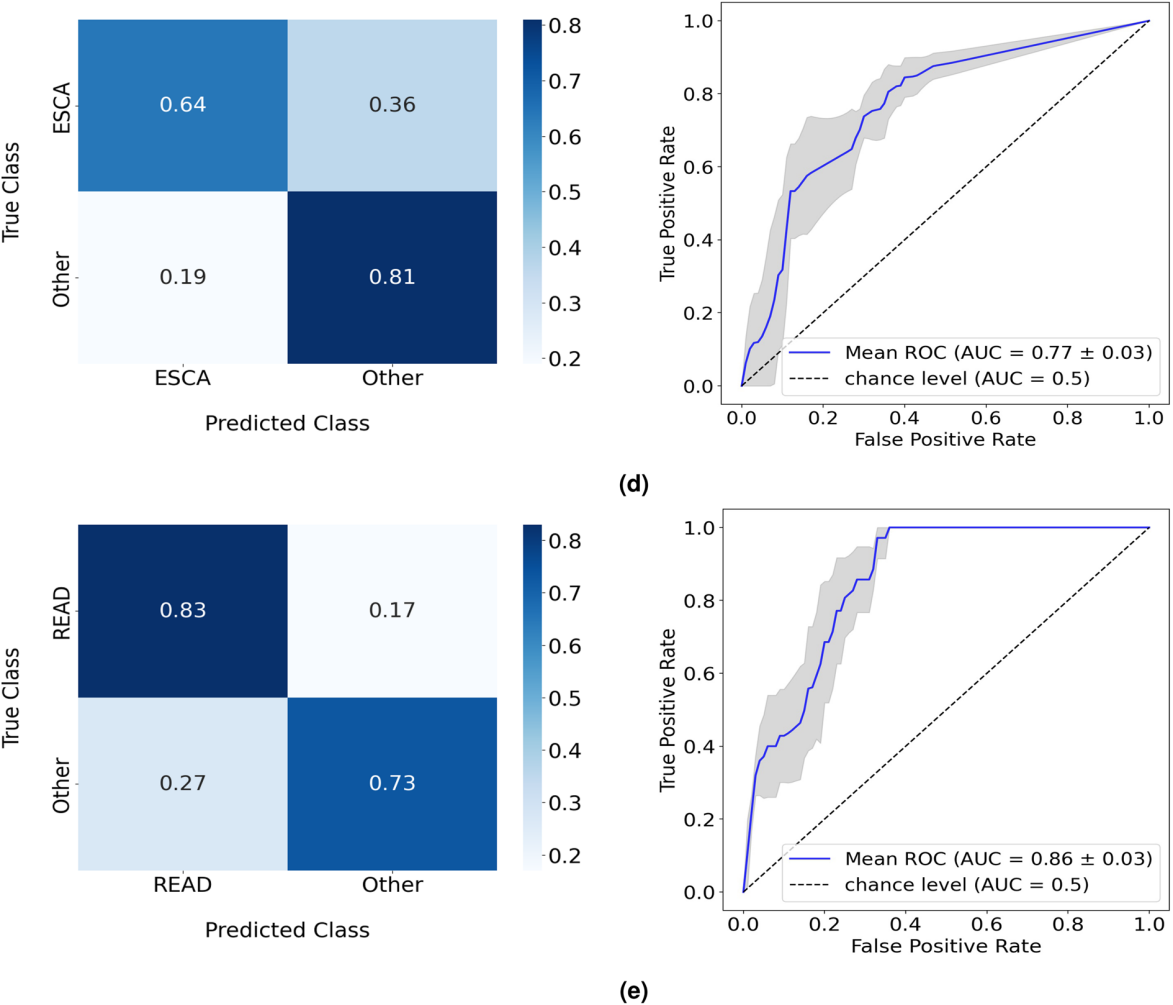
Normalized confusion matrices and corresponding ROC curves of the RF performances in the one-versus-all study, targeting (**a**) HNSC, (**b**) STAD, (**c**) COAD, (**d**) ESCA, and (**e**) READ cancer cases.

Results are summarized in Table 1 and can be analyzed in greater detail from Supplementary Tables S3 to S7. From the balanced accuracy of the RF models in the test split, the five cancers form two groups with distinct performances. HNSC, STAD, and COAD achieved balanced accuracy values ranging from 87% to 96% while for ESCA and READ, results show an increase in classification difficulty, with performances for both cases below 80%. These results are detailed in the corresponding confusion matrices (Fig. 3). The microbial composition of COAD was the most discriminative, with all samples of this type of cancer being correctly classified by the model. On the other hand, the confusion matrix from the ESCA analysis reveals that the major factor for the poor balanced accuracy of the RF model comes from the low accuracy of 64% when classifying ESCA samples, a significant difference in performance in comparison with the results from the other cancer types.

**Table 1:** One-versus-All study: best performance in the test split of the RF model for each cancer type. Results in more detail are found in Supplementary Tables S3 to S7. Results from 5-fold cross-validation are given as the balanced accuracy of the model in mean (%) ± standard deviation (%) format.

	Balanced accuracy
HNSC versus All	87.38 ± 2.19
STAD versus All	92.04 ± 1.02
COAD versus All	96.21 ± 0.42
ESCA versus All	72.35 ± 3.11
READ versus All	78.86 ± 6.15

In part, the results obtained from the one-versus-all study demonstrated that microbial data can be successfully applied to classify distinct cancer types independently with promising reliability. Nonetheless, there are also key discrepancies in performance among the different cancers. Although worse results coincide with classes with lower sample size, they may also suggest distinct degrees of complexity and the necessity to adapt ML implementations and the microbial information provided according to cancer type. Furthermore, the investigation conducted by Poore et al.^18^ encompassed a similar study of a supervised ML model on a one-versus-all approach discriminating, among others, these respective cancer types. Nevertheless, the large number of different cancer types also included in the analysis allied with the large discrepancy in sample size, in some cases having more than 10 times the number of samples provided by TCMA for specific cancer, invalidated the comparison of results.

### Study 2: three-class test

After assessing the performance of the RF when discriminating a specific type of cancer from the remaining, the 5 classes were grouped into 3 major ones: HNSC, STAD/ESCA, and CRC (COAD and READ).

**Figure 4:**
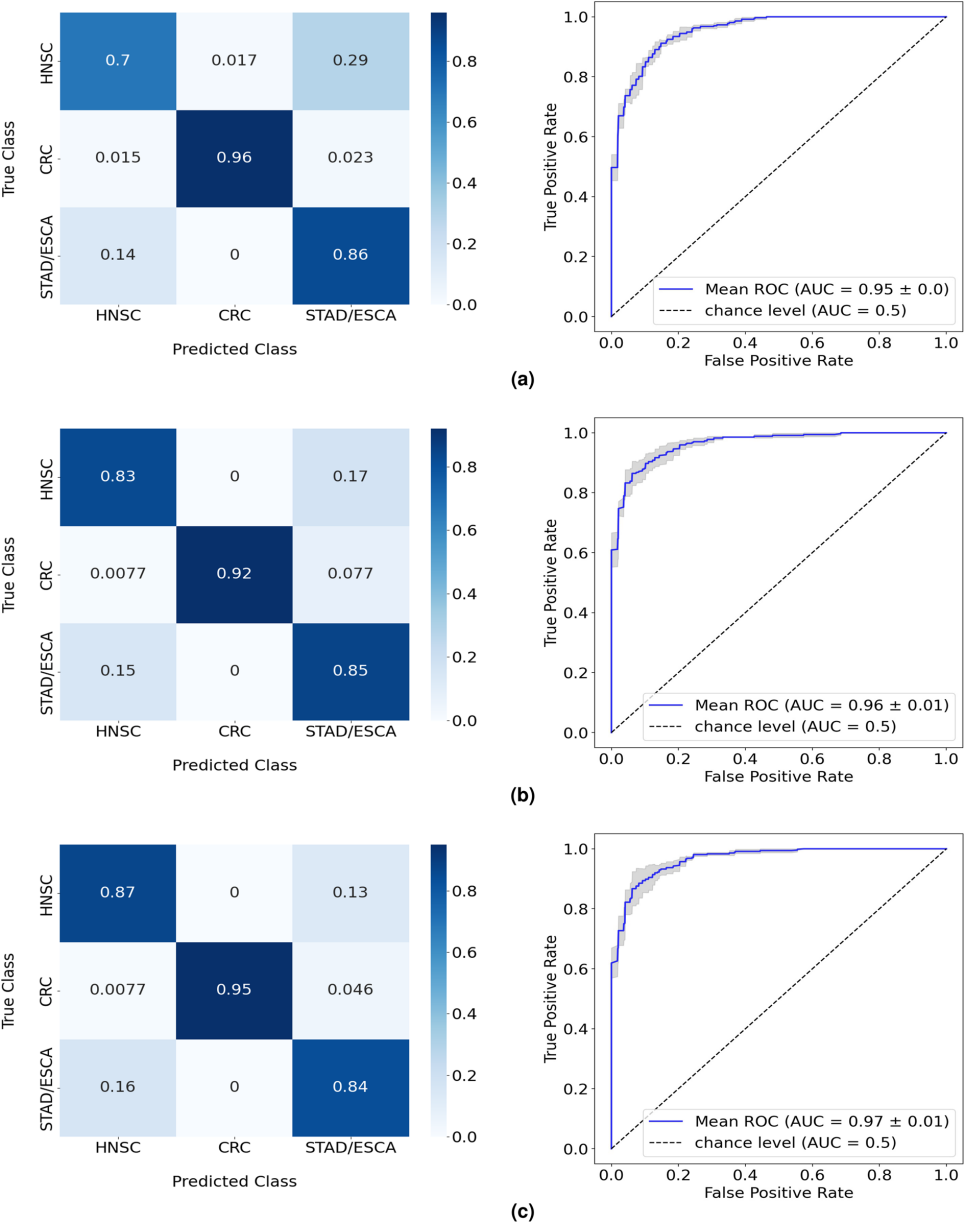
Normalized confusion matrices and corresponding ROC curves of the RF performances in the three-class test, with (**a**) the isolated implementation of RF, (**b**) the implementation of dimensionality reduction, and (**c**) the introduction of oversampling to the model alongside dimensionality reduction.

Across the various experiments, the RF model with dimensionality reduction and oversampling as auxiliary techniques returned the highest balanced accuracy of 88%, an increment of 4% in relation to the performance of the baseline model (Table 2). Consistent with the results from the one-versus-all study, CRC appeared to be the most easily separable among the 3 classes, with the RF maintaining accuracy levels above 90% (Fig. 4). The dimensionality reduction and oversampling techniques were mainly responsible for improving the performance of the RF when discriminating HNSC cancer cases, with its classification success rate increasing from 70% (Fig. 4a) to 87% (Fig. 4c).

**Table 2:** Performance in the test split of the RF model in the three-class study. Results in more detail are found in Supplementary Table S8. Results from 5-fold cross-validation are given as the balanced accuracy of the model in mean (%) ± standard deviation (%) format.

	Balanced accuracy
Experiment 1: RF	83.84 ± 3.06
Experiment 2: RF + Dimensionality reduction	86.13 ± 3.15
Experiment 3: RF + Feature engineering	86.25 ± 1.77
Experiment 4: RF + Dimensionality Reduction + Oversampling	88.28 ± 1.63
Experiment 5: RF + Feature engineering + oversampling	87.05 ± 2.30

Although being clear that the application of dimensionality reduction and oversampling to the dataset assisted the RF model in boosting its isolated performance, the improvements seen corresponded only to the HNSC class. With the classification of CRC cancer cases already hitting high accuracy levels, the experiments failed to bring advancements in performance in regard to the STAD/ESCA class. To further assess this situation, additional analyses were conducted where ESCA and STAD were treated as independent classes.

### Study 3: four-class test

Results from the previous study showcased the capability of the RF to classify with adequate accuracy HNSC, CRC, and STAD/ESCA classes. In greater detail though, dimensionality reduction and oversampling techniques failed to enhance the performance of the model when classifying STAD/ESCA cancer samples, solely boosting accuracy scores with respect to the HNSC class. To better comprehend the predictive limitations of the microbial data on a more granular level of specificity, STAD and ESCA samples were considered separately.

**Figure 5:**
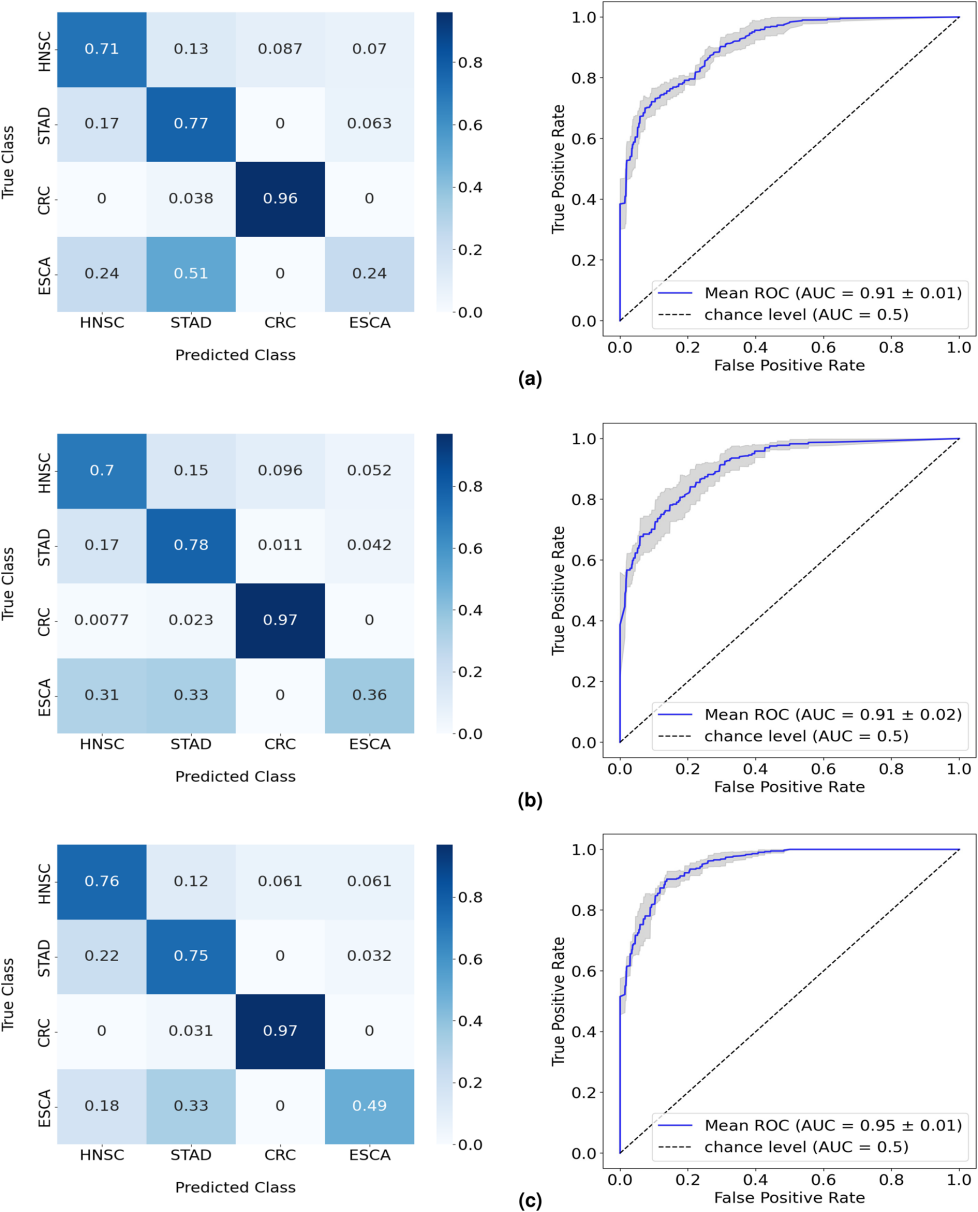
Normalized confusion matrices and corresponding ROC curves of the RF performances in the four-class test, with (**a**) the isolated implementation of RF, (**b**) the implementation of feature engineering, and (**c**) the introduction of oversampling to the model alongside feature engineering.

In this four-class classification problem, the RF achieved an overall performance of 74% balanced accuracy, with feature engineering and oversampling boosting the baseline result 7% (Table 3). The sample distribution for each class seen in Supplementary Figure S1 indicates a considerable imbalance between ESCA and the remaining classes and thus, oversampling was recognized as a necessary approach to obtain the highest performance from the model. Nevertheless, the significant decrease in overall performance compared with previous studies is evidently revealed by the confusion matrix (Fig. 5). The RF presented a low performance when discerning ESCA samples from the rest, only correctly classifying 50% of cases while wrongly predicting the remaining as HNSC or STAD (Fig. 5c). With the model revealing a moderate success rate of 75% in the classification of STAD cancer samples, ESCA is proving to be a critical limitation to the otherwise promising scores, intuition that was already supported by the results obtained from the one-versus-all study.

**Table 3:** Performance in the test split of the RF model in the four-class study. Results in more detail are found in Supplementary Table S9. Results from 5-fold cross-validation are given as the balanced accuracy of the model in mean (%) ± standard deviation (%) format.

	Balanced accuracy
Experiment 1: RF	67.21 ± 4.50
Experiment 2: RF + Dimensionality reduction	68.86 ± 1.82
Experiment 3: RF + Feature engineering	70.11 ± 4.76
Experiment 4: RF + Dimensionality reduction + Oversampling	72.80 ± 4.91
Experiment 5: RF + Feature engineering + Oversampling	74.06 ± 4.53

With a higher level of specificity in terms of cancer site, results show a clear loss in predictive power from the microbial data. In comparison to the three-class test, the RF reveals inferior performances when classifying HNSC cancer cases, and exhibits a distinct difficulty principally when discerning ESCA cases from STAD. On the other hand, the CRC class continues to stand out as the most discriminative, with accuracy scores above 95%. Since the CRC class is composed of COAD and READ cases, the following study was conducted with both cancer types treated separately.

### Study 4: five-class test

Throughout the previous studies, results have demonstrated a distinct capability of the microbial information to discriminate between high-performance CRC cancer cases from the remaining cancer types. With COAD and READ samples forming the CRC class, it is important to analyze with finer detail how these results translate to both cancers. In this context, it is also made a final assessment of the predictive power of the microbial data in the highest level of specificity possible in the investigation with a five-class classification problem.

**Figure 6:**
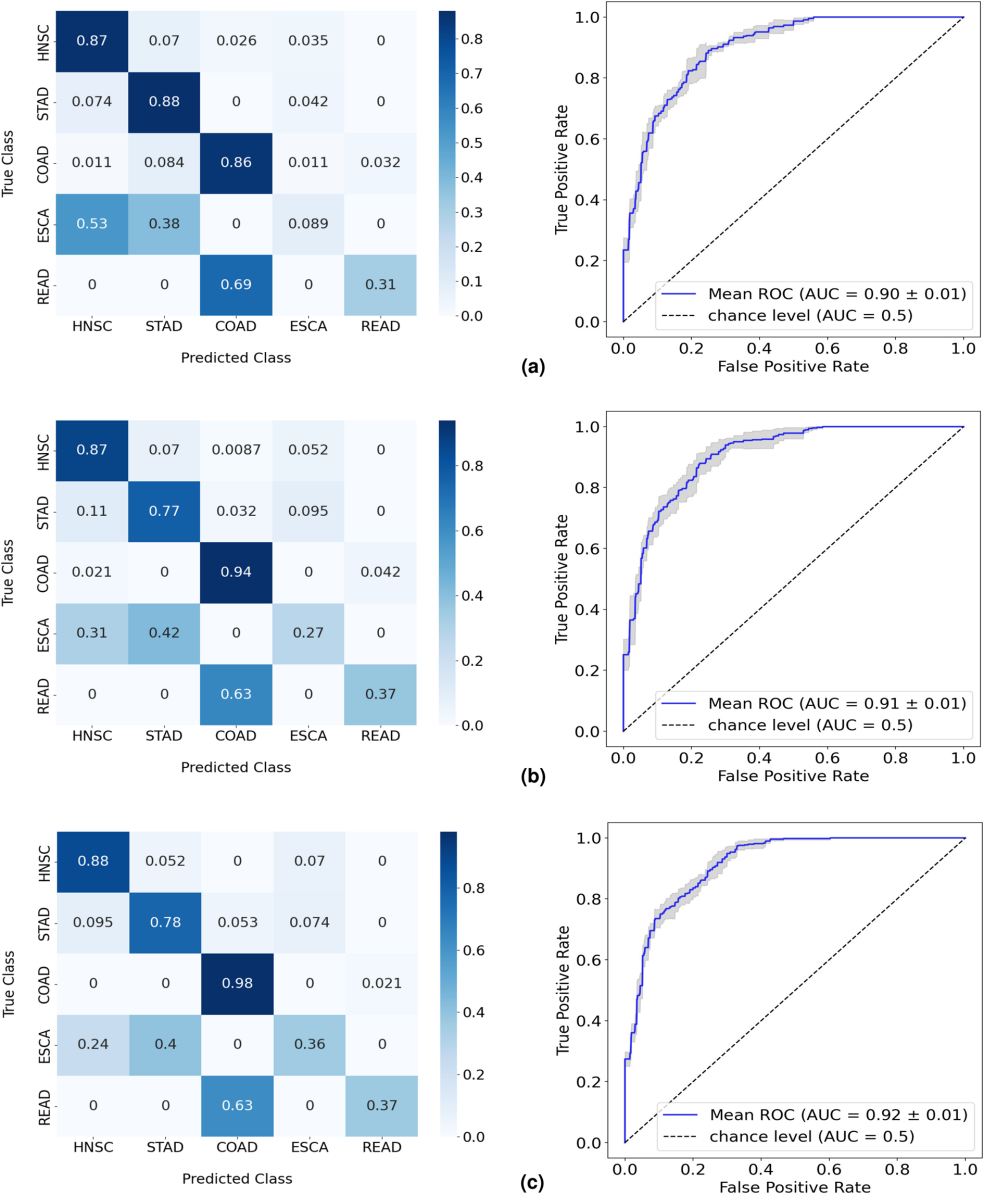
Normalized confusion matrices and corresponding ROC curves of the RF performances in the five-class test, with (**a**) the isolated implementation of RF, (**b**) the implementation of feature engineering, and (**c**) the introduction of oversampling to the model alongside feature engineering.

The RF model with feature engineering and oversampling achieved the highest overall performance of 67% balanced accuracy (Table 4). The sample distribution for each class shown in Supplementary Figure S1 indicates, beyond the ESCA class, a substantial imbalance between READ and the remaining cancers. Despite the promising results previously demonstrated when dealing with COAD and READ cases as a single class, Fig. 6 clearly shows that the RF is not able to distinguish READ samples from COAD, with 63% of cases incorrectly predicted as the latter (Fig. 6c). Furthermore, the model is also incapable of differentiating ESCA cases from HNSC and STAD, following the results of the four-class test. While it is expected that there may be a decrease in the performance of the model as the granularity level of analysis increases, this explanation alone does not fully account for the lower accuracy observed for HNSC in the four-class test compared to the five-class test (Figs. 5c, 6c). Similarly, a slight increase in performance from the four-class to the five-class test can be observed with STAD. One plausible explanation for these trends is that the inclusion of COAD and READ as independent classes in the five-class test improved the ability of the model to distinguish between HNSC and STAD, albeit at the cost of a less distinct separation between the preceding classes and ESCA. However, further investigation is warranted to better understand the factors influencing the differential performance across the four-class and five-class tests for HNSC and STAD.

**Table 4:** Performance in the test split of the RF model in the five-class study. Results in more detail are found in Supplementary Table S10. Results from 5-fold cross-validation are given as the balanced accuracy of the model in mean (%) ± standard deviation (%) format.

	Balanced accuracy
Experiment 1: RF	60.37 ± 2.93
Experiment 2: RF + Dimensionality Reduction	61.25 ± 4.39
Experiment 3: RF + Feature engineering	64.01 ± 2.25
Experiment 4: RF + Dimensionality reduction + Oversampling	60.38 ± 3.12
Experiment 5: RF + Feature engineering + Oversampling	67.31 ± 3.93

Overall, results indicate the existence of 2 cancer groups that differentiate in predictive performance. For HNSC, STAD, and COAD, the microbial data appeared to be a promising biomarker, with the microbiome of COAD standing out as the most discriminative class. On the contrary, there was a clear lack of capability of the RF models to classify ESCA and READ cancer cases based on microbial data. While READ samples were not accurately distinguished from COAD, ESCA cases were also, for the most part, improperly classified as HNSC or STAD.

### *Genera* contribution

The five-class test revealed two points of major fragility when applying the microbial information from the TCMA database as a predictor of cancer type, with the RF not being able to discern: (1) READ from COAD cases, and (2) ESCA from STAD and HNSC cases. In order to obtain a greater understanding of the poor results achieved in (1) and (2), an assessment was made on the contribution and distribution of the *genera* provided to the RF model for each situation. To accomplish this, SHAP^24^ (SHapley Additive exPlanations) was implemented as a method to try to explain the output of the RF and the contribution of each *genus* in the said output. Furthermore, box plots were constructed for various *genera* to assess their distribution across the different cancer types.

When isolating READ and COAD cases for analysis, Table 5 summarizes the *genera* that most contributed to the performance of the model for each cancer type, taking into account all predictions made by the model as well as separating the correct from the wrong predictions. When comparing both cancers, some *genera* are predominant, such as *Porphyromonas* and *Granulicatella*. Features introduced by feature engineering (in this case, components given by LDA) also appear for the two cancers, but due to their artificial nature and bias for improving the performance of RF models, they were not focused on evaluation. Supplementary Fig. S2a and S2b illustrate the box plots of the samples for *Porphyromonas* and *Granulicatella*, respectively. READ samples have a higher mean relative abundance of *Porphyromonas*. Nonetheless, there is a significant percentage of samples showing no abundance of both *genera* for READ and COAD cancer types. The similarity in the contribution and distribution of the *genera* that most contributed to the performance of the RF in this context highlights that only microbial information cannot discriminate cases with READ from cases with COAD.

**Table 5:** *Genera* with the most contribution on model predictions according to SHAP analysis for each cancer type (in descending order of contribution). Corresponding SHAP scores are provided for each feature.

	Overall		Correct predictions		Incorrect predictions	
COAD	*Porphyromonas*	(0.099)	*Porphyromonas*	(0.216)	*Porphyromonas*	(2.005)
	*Granulicatella*	(0.031)	*Akkermansia*	(0.033)	*Akkermansia*	(0.289)
	*Bacteroides*	(0.025)	*Granulicatella*	(0.031)	*Barnesiella*	(0.202)
	*Solobacterium*	(0.021)	*Streptococcus*	(0.029)	*Parabacteroides*	(0.115)
	*Fusobacterium*	(0.020)	*Alistipes*	(0.024)	*Ruminococcus*	(0.072)
READ	*Porphyromonas*	(0.294)	*Porphyromonas*	(0.965)	*Porphyromonas*	(0.209)
	*Granulicatella*	(0.117)	*Granulicatella*	(0.293)	*Parvimonas*	(0.156)
	*Streptococcus*	(0.070)	*Streptococcus*	(0.139)	*Bacteroides*	(0.035)
	*Solobacterium*	(0.042)	*Solobacterium*	(0.131)	*Akkermansia*	(0.022)
	*Parabacteroides*	(0.031)	*Campylobacter*	(0.111)	*Sutterella*	(0.022)
HNSC	*Helicobacter*	(0.236)	*Helicobacter*	(0.230)	*Leptotrichia*	(0.020)
	*Lactobacillus*	(0.044)	*Lactobacillus*	(0.040)	*Alloprevotella*	(0.011)
	*Fusobacterium*	(0.011)	*Fusobacterium*	(0.013)	*Acinetobacter*	(0.009)
	*Leptotrichia*	(0.006)	*Alloprevotella*	(0.005)	*Prevotella*	(0.008)
	*Alloprevotella*	(0.005)	*Clostridium*	(0.005)	*Abiotrophia*	(0.008)
STAD	*Helicobacter*	(0.336)	*Helicobacter*	(0.433)	*Helicobacter*	(0.072)
	*Lactobacillus*	(0.042)	*Capnocytophaga*	(0.019)	*Prevotella*	(0.020)
	*Veillonella*	(0.017)	*Lactobacillus*	(0.013)	*Clostridium*	(0.013)
	*Fusobacterium*	(0.012)	*Clostridium*	(0.009)	*Capnocytophaga*	(0.012)
	*Prevotella*	(0.012)	*Fusobacterium*	(0.009)	*Actinomyces*	(0.009)
ESCA	*Helicobacter*	(0.131)	*Helicobacter*	(0.203)	*Lactobacillus*	(0.066)
	*Streptococcus*	(0.042)	*Lactobacillus*	(0.097)	*Streptococcus*	(0.055)
	*Prevotella*	(0.031)	*Acinetobacter*	(0.034)	*Prevotella*	(0.038)
	*Lactobacillus*	(0.030)	*Capnocytophaga*	(0.018)	*Atopobium*	(0.026)
	*Aloprevotella*	(0.022)	*Prevotella*	(0.009)	*Abiotrophia*	(0.011)

Similarly, there is an overlap on *genera* contribution for HNSC, ESCA, and STAD (Table 5). In addition to the complementary features provided by LDA, *genera* such as *Helicobacter*, *Lactobacillus*, and *Fusobacterium* are seen across the various cancers, an indicator of their analogous microbial data. Furthermore, box plots of *genera* including *Fusobacterium* (Supplementary Fig. S2c), *Porphyromonas* (Supplementary Fig. S2d), and *Capnocytophaga* (Supplementary Fig. S2e) reveal a closer relationship between ESCA and STAD cancers rather than between ESCA and HNSC. This analysis is congruent with the results of the five-class test that showed a percentage of ESCA cases incorrectly classified as STAD greater than the percentage of cases incorrectly predicted as HNSC. Identically to the conclusions taken for READ and COAD, the resemblance in contribution and distribution of various *genera* supports the lack of predictive power presented by the microbial information when discriminating ESCA samples from STAD and HNSC.

The SHAP analysis conducted in this study played a crucial role in quantifying and identifying important microbial *genera* based on their impact on the model’s performance. Notably, *Granulicatella* and *Porphyromonas* emerged as important *genera* when distinguishing between COAD and READ cancer samples, which aligns with previous research highlighting *Granulicatella*’s strong association and enrichment in colorectal cancer tissues^25,26^, as well as *Porphyromonas*’ role in promoting colorectal tumor progression by recruiting myeloid cells and establishing an inflammatory tumor microenvironment, along with promoting colorectal cancer cell proliferation through MAPK/ERK signaling pathway activation^27,28^. Furthermore, other *genera* such as *Helicobacter*, *Lactobacillus*, and *Fusobacterium* were also identified as significant when discriminating HNSC, STAD, and ESCA cancer samples. While Helicobacter has been associated with head and neck cancer^29^, its presence in these tumors is controverse^30^. In the case of gastric cancer, however, there is no doubt on the causal relationship between Helicobacter (pylori) and non-cardia gastric cancer^31^. Additionally, a protective effect for H. pylori infection has been found for esophageal adenocarcinoma, whereas no associations between this infection and esophageal squamous cell carcinoma were identified^32^. *Lactobacillus* overgrowth has been linked to the development of gastric cancer, attributed to its metabolic products and alterations in the microbial community, and its abundance has shown a close association with the development of esophageal cancer^33–35^. *Fusobacterium* can promote cancer through various mechanisms, including cell proliferation, cellular invasion, chronic inflammation induction, and immune evasion^36^. Its higher presence and abundance in head and neck cancer samples, compared to non-cancer samples, suggest its potential role in the development of this cancer^37^. *Fusobacterium* infection has also been associated with increased mutations and progression of gastric cancer, as well as enhanced growth ability of esophageal squamous cell carcinoma cells, contributing to tumor progression^38,39^.

### Limitations and future work

This work represents only a small perspective on the overall scope of ML applicability to study the microbiome-cancer relationship. There are additional approaches that could improve, progress, and branch out of this type of study. Some of these approaches are specified as follows:

- Increasing the number of samples, mainly of READ and ESCA cancers, could serve as a direct measure to improve the performance of the ML models when predicting these classes. The lack of samples relative to the remaining cancer types might have been a major reason for the poor results obtained. In addition to the small sample size, the ESCA cancer type is a heterogeneous group of cancers, encompassing squamous cell carcinomas and adenocarcinomas, which may be located in the proximal or distal esophagus, and which may have distinct microbial signatures that compromised the ML model discrimination power for this type of cancer;
- ML modeling with microbial information at lower taxonomic levels could bring new insights into the analysis. This work was performed using microbial data at the *genus* level, but other possibilities could be implemented, including the use of data at the *species* or strain level;
- An independent dataset serves as the optimal means to assess the robustness of predictive models. Unfortunately, we currently lack access to an independent dataset for testing purposes. As a viable alternative, cross-validation was employed and a separate, previously unseen subset of the available data was used for testing to evaluate the performance of the models;
- The primary focus of the current study was exploring the potential of microbiome data in predicting cancer types. However, it is essential to acknowledge that clinical assessments incorporate various sources of data. Therefore, future predictive models should encompass clinical data, including age, gender, BMI, smoking status, and other relevant information pertaining to cancer patients. By integrating these additional factors, a more comprehensive analysis can be conducted, facilitating a deeper understanding of the pathological changes taking place;
- The study was limited by the absence of cancer staging information in the available sample metadata, which prevented a comprehensive examination of the microbial signatures associated with different stages of cancer. However, by focusing on early-stage cancers, researchers can gain more meaningful insights into the microbial changes and signatures specifically associated with the initial development of cancer. Addressing this limitation will contribute to a better understanding of the role of the human microbiome in cancer progression.

## Conclusions

The main goal of this work was to develop an ML approach to discriminate different types of cancer based on the analysis of their specific microbial information. For this purpose, RF models were trained for the classification of HNSC, STAD, COAD, ESCA, and READ cancers, with dimensionality reduction and oversampling techniques proving to assist in the improvement of performance. Through the studies conducted, an assessment was made on the evolution of the predictive power of the microbial information with the increase in the specificity degree of the cancer site. Essentially, confusion matrices revealed promising performances when predicting HNSC, STAD, and COAD cases, with the latter being associated with outstanding accuracy scores. However, there was an increased difficulty in the capability of the RF models to differentiate ESCA from HNSC and STAD cases, and READ from COAD cases, with acceptable results, coinciding with a reduced number of samples for both cancers in comparison to others. By analyzing the distribution and contribution to the predictions of *genera* associated with ESCA and READ cancer types, the similarities found between READ and COAD, and between ESCA, STAD, and HNSC are evidence of the poor performances achieved when classifying READ and ESCA cases. Despite this limitation, the use of ML to analyze cancer microbiome data shows that it has the potential to aid in the development of new strategies for cancer detection and prevention, possibly finding new relationships unknown yet and ultimately reducing the burden of the disease.

## Supplementary Information

Supplementary Information.

## Data Availability

The data that supports the findings of this study are openly available in The Cancer Microbiome Atlas at https://tcma.pratt.duke.edu.
