# Supplementary material for: Machine learning-based approaches for cancer prediction using microbiome data
Source: Sci Rep. 2023 Jul 21;13:11821. doi: 10.1038/s41598-023-38670-0 (PMC10362018; doi:10.1038/s41598-023-38670-0)
Supplement: Supplementary file 1 — Supplementary Information. [file 41598_2023_38670_MOESM1_ESM.pdf]

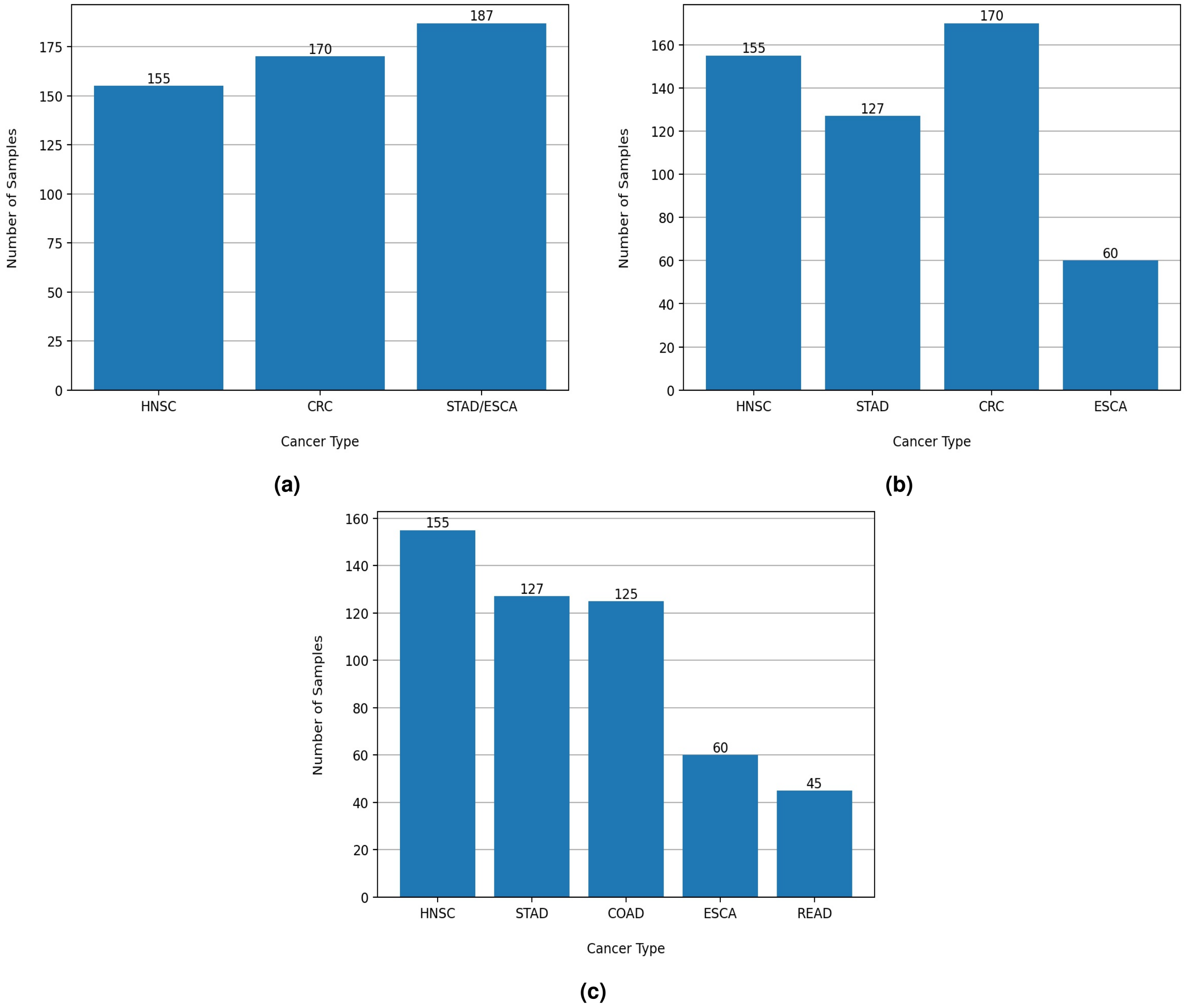

Figure S1. Sample distribution of the dataset according to cancer type for the: (a) three-class test with HNSC, CRC, and STAD/ESCA; (b) four-class test with HNSC, STAD, CRC, and ESCA; (c) five-class test with HNSC, STAD, CRC, ESCA, and READ.

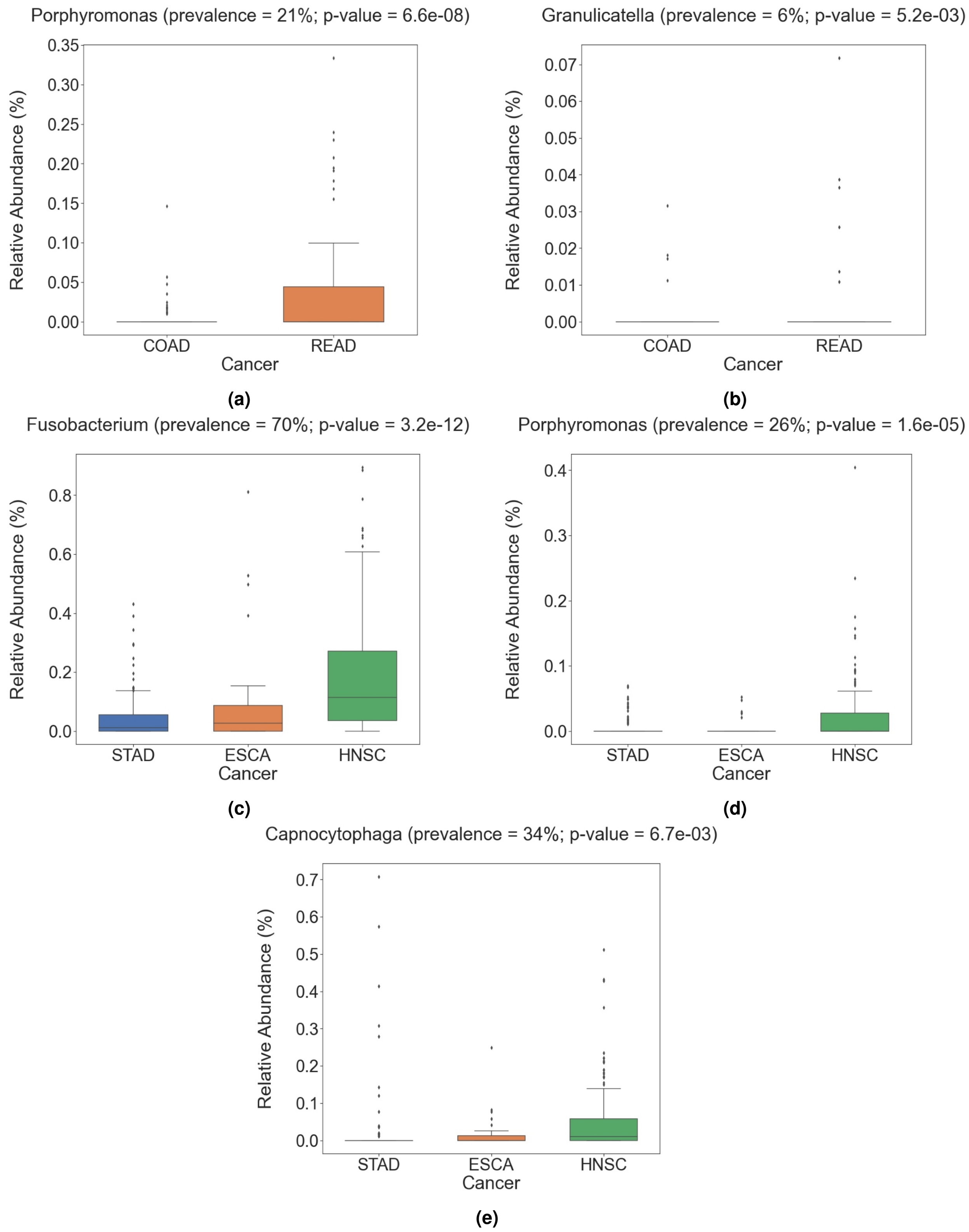

Figure S2. Box plots illustrating feature distribution for: comparing COAD and READ cancer cases through (a) *Porphyromonas* and (b) *Granulicatella genera*; comparing HNSC, STAD, and ESCA cancer cases through (c) *Fusobacterium*, (d) *Porphyromonas*, and (e) *Capnocytophaga genera*.

Simonsiella	*Treponema*	*Campylobacter*	*Helicobacter*
Paracoccus	*Comamonas*	*Pseudomonas*	*Xanthomonas*
Agrobacterium	*Bradyrhizobium*	*Acinetobacter*	*Neisseria*
Eikenella	*Citrobacter*	*Enterobacter*	*Escherichia*
Klebsiella	*Shigella*	*Haemophilus*	*Bacteroides*
Butyrivibrio	*Porphyromonas*	*Prevotella*	*Roseburia*
Fusobacterium	*Desulfovibrio*	*Megasphaera*	*Selenomonas*
Capnocytophaga	*Peptostreptococcus*	*Ruminococcus*	*Staphylococcus*
Streptococcus	*Enterococcus*	*Gemella*	*Atopobium*
Clostridium	*Lactobacillus*	*Actinomyces*	*Bifidobacterium*
Corynebacterium	*Eubacterium*	*Propionibacterium*	*Mycobacterium*
Gordonia	*Mycoplasma*	*Thermosipho*	*Gardnerella*
Lachnospira	*Veillonella*	*Leptotrichia*	*Rothia*
Kingella	*Phascolarctobacterium*	*Coprococcus*	*Bilophila*
Dialister	*Sutterella*	*Tissierella*	*Johnsonella*
Catonella	*Filifactor*	*Abiotrophia*	*Lautropia*
Mitsuokella	*Chryseobacterium*	*Centipeda*	*Eggerthella*
Cryptobacterium	*Pedobacter*	*Mogibacterium*	*Coprobacillus*
Collinsella	*Ensifer*	*Pseudoramibacter*	*Granulicatella*
Bulleidia	*Solobacterium*	*Olsenella*	*Catenibacterium*
Anaeroglobus	*Peptoniphilus*	*Anaerococcus*	*Sneathia*
Shuttleworthia	*Varibaculum*	*Dorea*	*Tannerella*
Scardovia	*Faecalibacterium*	*Ottowia*	*Alistipes*
Akkermansia	*Marvinbryantia*	*Oribacterium*	*Odoribacter*
Subdoligranulum	*Parabacteroides*	*Gulbenkiania*	*Barnesiella*
Aggregatibacter	*Alloscardovia*	*Adlercreutzia*	*Oscillibacter*
Parvimonas	*Blautia*	*Butyricimonas*	*Paraprevotella*
Pyramidobacter	*Lachnoanaerobaculum*	*Stomatobaculum*	*Eggerthia*
Alloprevotella	*Lelliottia*	*Coprobacter*	*Intestinimonas*
Fusicatenibacter	*Lachnoclostridium*	*Tyzzerella*	*Faecalitalea*
Holdemanella	*Mageeibacillus*	*Hungatella*	*Pseudopropionibacterium*
Peptoanaerobacter	*Emergencia*	*Prevotellamassilia*	*Criibacterium*
Fournierella	*Negativibacillus*	*Duodenibacillus*	

Table S1. List of 131 genera included as features in the ML models, excluding genera absent in any sample.

**Hyper-parameter**	**Value Range**
n_estimators	1 to 400
criterion	Gini impurity or Shannon information gain
min_samples_split	2 to 100
min_samples_leaf	1 to 20
max_depth	1 to 50 when specified
max_features	1 to maximum number of features

Table S2. Hyper-parameters and their value range used for the tuning of RF models.

HNSC-*vs*-All

		No. of Features	Train	Validation	Test
RF		131	89.06 ± 1.05	83.69 ± 5.46	80.78 ± 2.22
RF + Dimensionality Reduction	SPCA	131 → 100	84.00 ± 1.22	79.64 ± 4.79	81.72 ± 2.23
	NMF	131 → 70	88.70 ± 1.47	84.14 ± 3.19	79.87 ± 2.09
	LDA	131 → 1	93.55 ± 0.99	80.99 ± 5.35	75.28 ± 2.93
RF + Feature Engineering	SPCA	131 → 135	88.91 ± 0.83	83.74 ± 4.59	82.83 ± 3.09
	NMF	131 → 139	**88.00** ± **0.68**	**84.23** ± **3.87**	**83.16** ± **1.99**
	LDA	131 → 132	94.19 ± 1.15	82.53 ± 5.55	78.50 ± 5.88

			Equaling all classes to the majority class			Oversampling all classes by 50% relatively to the majority class			Oversampling all classes by 100% relatively to the majority class		
			Train	Validation	Test	Train	Validation	Test	Train	Validation	Test
RF + Dimensionality Reduction + Oversampling	Random Oversampler	100	89.89 ± 1.03	80.51 ± 4.47	80.37 ± 1.37	93.22 ± 0.45	81.11 ± 4.57	83.66 ± 3.09	95.14 ± 0.30	81.15 ± 5.11	83.16 ± 1.88
	SVM SMOTE		89.10 ± 0.47	83.59 ± 1.93	84.04 ± 3.75	91.85 ± 0.57	82.98 ± 5.60	86.63 ± 3.92	92.83 ± 0.53	83.36 ± 3.05	86.70 ± 3.87
RF + Feature Engineering + Oversampling	Random Oversampler	139	93.98 ± 0.29	84.24 ± 4.00	84.60 ± 0.91	95.32 ± 0.42	83.01 ± 5.83	83.77 ± 2.06	96.74 ± 0.14	83.66 ± 4.35	86.25 ± 0.87
	SVM SMOTE		92.86 ± 0.93	85.07 ± 5.98	86.77 ± 2.70	**94.74** ± **0.55**	**86.32** ± **3.78**	**87.38** ± **2.19**	96.49 ± 0.20	85.94 ± 5.88	84.77 ± 1.75

Table S3. Performance of the RF model in the HNSC-*vs*-all study with the isolated implementation of RF, the implementation of dimensionality reduction / feature engineering, and the introduction of oversampling to the model. Results from 5-fold cross-validation are given as the balanced accuracy of the model in mean (%) ± standard deviation (%) format.

STAD-*vs*-All

		No. of Features	Train	Validation	Test
RF		131	86.74 ± 1.85	79.91 ± 3.73	82.84 ± 2.74
RF + Dimensionality Reduction	SPCA	131 → 60	89.71 ± 2.25	78.18 ± 5.53	78.49 ± 6.70
	NMF	131 → 60	94.15 ± 1.10	83.25 ± 5.61	78.70 ± 6.71
	LDA	131 → 1	93.10 ± 1.36	80.22 ± 3.31	79.95 ± 4.60
RF + Feature Engineering	SPCA	131 → 163	89.99 ± 2.52	80.69 ± 2.86	82.33 ± 4.14
	NMF	131 → 139	91.68 ± 1.69	82.84 ± 4.23	83.02 ± 4.35
	LDA	131 → 132	**95.57** ± **0.32**	**82.76** ± **2.63**	**86.97** ± **2.07**

			Equaling all classes to the majority class			Oversampling all classes by 50% relatively to the majority class			Oversampling all classes by 100% relatively to the majority class		
			Train	Validation	Test	Train	Validation	Test	Train	Validation	Test
RF + Dimensionality Reduction + Oversampling	Random Oversampler	1	94.88 ± 0.85	82.85 ± 4.32	87.19 ± 2.43	94.55 ± 0.71	84.67 ± 4.38	84.39 ± 2.64	94.43 ± 0.63	82.80 ± 5.08	86.14 ± 2.21
	SVM SMOTE		93.58 ± 0.62	85.46 ± 2.97	86.16 ± 2.46	90.73 ± 1.51	85.09 ± 4.13	85.42 ± 3.48	87.31 ± 1.74	84.20 ± 2.95	84.95 ± 2.57
RF + Feature Engineering + Oversampling	Random Oversampler	132	97.94 ± 0.70	85.69 ± 2.82	89.60 ± 4.31	98.32 ± 0.47	83.88 ± 2.51	88.71 ± 2.34	98.91 ± 0.22	83.51 ± 2.73	88.18 ± 4.77
	SVM SMOTE		97.84 ± 0.39	87.71 ± 1.99	89.60 ± 3.06	**97.99** ± **0.29**	**86.77** ± **1.94**	**92.04** ± **1.02**	98.06 ± 0.26	87.40 ± 1.47	88.73 ± 1.46

Table S4. Performance of the RF model in the STAD-*vs*-all study with the isolated implementation of RF, the implementation of dimensionality reduction / feature engineering, and the introduction of oversampling to the model. Results from 5-fold cross-validation are given as the balanced accuracy of the model in mean (%) ± standard deviation (%) format.

COAD-*vs*-All

		No. of Features	Train	Validation	Test
RF		131	94.34 ± 0.74	92.30 ± 2.81	93.54 ± 1.05
RF + Dimensionality Reduction	SPCA	131 → 60	92.50 ± 0.75	91.84 ± 3.08	91.10 ± 1.40
	NMF	131 → 80	95.02 ± 1.46	91.98 ± 2.28	91.97 ± 2.68
	LDA	131 → 1	**92.19 ± 1.32**	**88.72 ± 3.68**	**94.26 ± 3.41**
RF + Feature Engineering	SPCA	131 → 135	94.14 ± 1.08	91.98 ± 3.93	93.88 ± 1.92
	NMF	131 → 163	94.05 ± 0.75	91.31 ± 4.42	89.16 ± 1.19
	LDA	131 → 132	94.05 ± 1.00	91.46 ± 4.25	93.36 ± 4.32

			Equaling all classes to the majority class			Oversampling all classes by 50% relatively to the majority class			Oversampling all classes by 100% relatively to the majority class		
			Train	Validation	Test	Train	Validation	Test	Train	Validation	Test
RF + Dimensionality Reduction + Oversampling	Random Oversampler	1	92.55 ± 0.86	92.08 ± 2.12	96.38 ± 0.34	81.09 ± 1.16	91.45 ± 2.35	95.68 ± 1.23	93.57 ± 0.67	90.99 ± 2.42	95.85 ± 1.02
	SVM SMOTE		**93.31 ± 0.40**	**92.23 ± 2.52**	**96.21 ± 0.42**	86.07 ± 1.10	91.23 ± 3.49	95.84 ± 1.42	85.49 ± 1.40	90.93 ± 3.98	94.80 ± 2.00
RF + Feature Engineering + Oversampling	Random Oversampler	132	95.48 ± 0.27	91.83 ± 2.87	94.42 ± 2.97	96.47 ± 0.61	90.91 ± 2.49	93.72 ± 2.51	97.21 ± 0.36	91.67 ± 2.56	95.47 ± 0.34
	SVM SMOTE		95.40 ± 0.30	93.11 ± 3.02	96.00 ± 1.19	93.58 ± 0.79	93.06 ± 2.49	96.88 ± 1.06	94.05 ± 0.57	92.17 ± 2.37	94.78 ± 1.99

Table S5. Performance of the RF model in the COAD-*vs*-all study with the isolated implementation of RF, the implementation of dimensionality reduction / feature engineering, and the introduction of oversampling to the model. Results from 5-fold cross-validation are given as the balanced accuracy of the model in mean (%) ± standard deviation (%) format.

ESCA-*vs*-All

		No. of Features	Train	Validation	Test
RF		131	84.45 ± 5.41	72.38 ± 8.81	61.37 ± 6.42
RF + Dimensionality Reduction	SPCA	131 → 30	76.36 ± 2.78	62.95 ± 3.62	57.68 ± 8.33
	NMF	131 → 90	83.75 ± 3.83	69.99 ± 3.41	61.31 ± 6.58
	LDA	131 → 1	91.07 ± 2.77	67.34 ± 3.38	61.60 ± 5.29
RF + Feature Engineering	SPCA	131 → 147	81.94 ± 5.99	69.00 ± 9.63	61.81 ± 8.90
	NMF	131 → 163	80.83 ± 2.09	70.72 ± 10.18	55.59 ± 7.58
	LDA	131 → 132	**90.40 ± 2.76**	**69.30 ± 8.60**	**64.41 ± 3.27**

			Equaling all classes to the majority class			Oversampling all classes by 50% relatively to the majority class			Oversampling all classes by 100% relatively to the majority class		
			Train	Validation	Test	Train	Validation	Test	Train	Validation	Test
RF + Dimensionality Reduction + Oversampling	Random Oversampler	90	96.03 ± 0.91	66.92 ± 9.98	67.32 ± 6.03	97.20 ± 0.64	71.21 ± 8.44	69.72 ± 5.46	98.81 ± 0.43	67.65 ± 11.66	64.53 ± 7.87
	SVM SMOTE		92.46 ± 0.66	75.76 ± 8.08	68.82 ± 4.58	92.73 ± 2.00	76.16 ± 6.62	65.85 ± 6.49	95.12 ± 0.98	75.81 ± 5.32	65.78 ± 6.47
RF + Feature Engineering + Oversampling	Random Oversampler	132	96.84 ± 0.80	72.83 ± 8.83	58.87 ± 5.34	97.59 ± 0.26	74.38 ± 6.90	57.89 ± 5.19	98.66 ± 0.15	73.72 ± 9.89	69.71 ± 5.77
	SVM SMOTE		**96.91 ± 0.57**	**80.93 ± 5.80**	**72.35 ± 3.11**	95.19 ± 0.86	79.02 ± 8.65	68.15 ± 6.17	96.44 ± 0.65	79.29 ± 8.84	66.60 ± 3.93

Table S6. Performance of the RF model in the ESCA-*vs*-all study with the isolated implementation of RF, the implementation of dimensionality reduction / feature engineering, and the introduction of oversampling to the model. Results from 5-fold cross-validation are given as the balanced accuracy of the model in mean (%) ± standard deviation (%) format.

READ-*vs*-All

		No. of Features	Train	Validation	Test
RF		131	78.01 ± 1.11	69.12 ± 5.40	56.43 ± 10.22
RF + Dimensionality Reduction	SPCA	131 → 30	72.44 ± 2.00	68.74 ± 6.71	59.86 ± 9.99
	NMF	131 → 90	**84.04** ± **4.44**	**77.13** ± **7.79**	**69.29** ± **6.23**
	LDA	131 → 1	87.22 ± 3.26	62.67 ± 8.16	60.86 ± 3.33
RF + Feature Engineering	SPCA	131 → 135	77.29 ± 2.77	71.62 ± 8.38	58.43 ± 12.03
	NMF	131 → 135	77.15 ± 1.80	73.23 ± 9.70	55.71 ± 0.64
	LDA	131 → 132	83.01 ± 3.88	64.13 ± 7.48	60.57 ± 3.54

			Equaling all classes to the majority class			Oversampling all classes by 50% relatively to the majority class			Oversampling all classes by 100% relatively to the majority class		
			Train	Validation	Test	Train	Validation	Test	Train	Validation	Test
RF + Dimensionality Reduction + Oversampling	Random Oversampler	90	**98.05** ± **0.25**	**82.24** ± **5.89**	**78.86** ± **6.15**	98.00 ± 0.31	65.77 ± 16.44	60.86 ± 5.77	98.68 ± 0.18	74.34 ± 7.99	64.43 ± 5.86
	SVM SMOTE		98.27 ± 0.39	83.41 ± 5.93	71.57 ± 5.67	98.51 ± 0.37	80.42 ± 8.61	66.57 ± 4.77	99.34 ± 0.32	80.62 ± 8.19	71.86 ± 6.02
RF + Feature Engineering + Oversampling	Random Oversampler	132	97.67 ± 0.18	69.56 ± 8.13	66.71 ± 6.48	98.44 ± 0.80	69.81 ± 8.21	68.29 ± 4.77	98.47 ± 0.70	67.33 ± 8.07	75.29 ± 12.27
	SVM SMOTE		96.86 ± 1.43	75.70 ± 7.79	66.86 ± 7.47	97.84 ± 0.23	78.58 ± 8.39	67.57 ± 5.65	98.03 ± 0.34	79.78 ± 13.22	73.29 ± 7.78

Table S7. Performance of the RF model in the READ-*vs*-all study with the isolated implementation of RF, the implementation of dimensionality reduction / feature engineering, and the introduction of oversampling to the model. Results from 5-fold cross-validation are given as the balanced accuracy of the model in mean (%) ± standard deviation (%) format.

Study 2: Three-class test

		No. of Features	Train	Validation	Test
RF		131	88.85 ± 0.38	86.33 ± 2.35	83.84 ± 3.06
RF + Dimensionality Reduction	SPCA	131 → 30	87.82 ± 0.50	84.29 ± 3.83	83.22 ± 2.72
	NMF	131 → 40	90.09 ± 0.85	86.11 ± 2.09	84.53 ± 4.24
	LDA	131 → 2	**89.55 ± 0.98**	**82.84 ± 4.17**	**86.13 ± 3.15**
RF + Feature Engineering	SPCA	131 → 147	88.33 ± 0.89	84.74 ± 2.73	84.19 ± 1.80
	NMF	131 → 163	89.71 ± 1.30	86.08 ± 2.76	82.50 ± 2.71
	LDA	131 → 133	94.08 ± 0.39	86.10 ± 4.45	86.25 ± 1.77

			Equaling all classes to the majority class			Oversampling all classes by 50% relatively to the majority class			Oversampling all classes by 100% relatively to the majority class		
			Train	Validation	Test	Train	Validation	Test	Train	Validation	Test
RF + Dimensionality Reduction + Oversampling	Random Oversampler	2	87.79 ± 1.65	83.32 ± 4.95	87.17 ± 2.33	89.33 ± 0.60	83.44 ± 3.23	87.20 ± 2.09	80.18 ± 0.74	83.80 ± 3.98	86.36 ± 0.88
	SVM SMOTE		**89.05 ± 0.27**	**84.10 ± 4.51**	**88.28 ± 1.63**	89.16 ± 1.21	84.78 ± 3.69	87.10 ± 2.53	88.90 ± 1.16	84.64 ± 4.24	87.59 ± 1.10
RF + Feature Engineering + Oversampling	Random Oversampler	133	95.70 ± 0.48	87.81 ± 3.60	86.65 ± 2.14	97.05 ± 0.37	84.47 ± 2.96	85.29 ± 1.48	97.72 ± 0.29	88.40 ± 2.80	85.80 ± 2.27
	SVM SMOTE		94.47 ± 1.00	88.54 ± 1.98	86.69 ± 1.48	95.82 ± 0.57	89.12 ± 2.57	87.05 ± 2.30	95.54 ± 0.52	89.07 ± 4.09	87.20 ± 2.29

Table S8. Performance of the RF model in the three-class test with the isolated implementation of RF, the implementation of dimensionality reduction / feature engineering, and the introduction of oversampling to the model. Results from 5-fold cross-validation are given as the balanced accuracy of the model in mean (%) ± standard deviation (%) format.

Study 3: Four-class test

		No. of Features	Train	Validation	Test
RF		131	89.33 ± 0.86	73.19 ± 5.37	67.21 ± 4.50
RF + Dimensionality Reduction	SPCA	131 → 60	87.30 ± 1.35	68.37 ± 3.60	68.86 ± 1.82
	NMF	131 → 50	89.73 ± 1.24	71.20 ± 2.23	63.75 ± 3.53
	LDA	131 → 3	87.78 ± 1.13	71.58 ± 3.40	62.99 ± 3.70
RF + Feature Engineering	SPCA	131 → 135	89.20 ± 1.51	72.20 ± 4.06	64.98 ± 2.87
	NMF	131 → 195	**90.82** ± **0.94**	**71.90** ± **4.66**	**70.11** ± **4.76**
	LDA	131 → 134	92.69 ± 0.98	75.58 ± 5.33	63.22 ± 1.59

			Equaling all classes to the majority class			Oversampling all classes by 50% relatively to the majority class			Oversampling all classes by 100% relatively to the majority class		
			Train	Validation	Test	Train	Validation	Test	Train	Validation	Test
RF + Dimensionality Reduction + Oversampling	Random Oversampler	60	93.32 ± 1.27	67.91 ± 3.85	70.78 ± 2.11	97.12 ± 0.43	68.37 ± 4.63	66.59 ± 3.71	94.80 ± 0.66	66.95 ± 6.75	70.39 ± 5.79
	SVM SMOTE		93.75 ± 1.15	71.61 ± 6.34	70.21 ± 3.52	94.84 ± 0.70	71.09 ± 4.92	72.80 ± 4.91	95.12 ± 1.34	73.90 ± 8.12	70.37 ± 2.44
RF + Feature Engineering + Oversampling	Random Oversampler	195	95.09 ± 0.67	72.63 ± 2.98	69.93 ± 2.57	94.80 ± 0.99	72.76 ± 6.99	71.92 ± 3.07	**96.24** ± **0.59**	**70.12** ± **4.48**	**74.06** ± **4.53**
	SVM SMOTE		94.59 ± 1.34	77.73 ± 5.19	65.34 ± 4.38	95.57 ± 0.87	77.01 ± 3.18	69.09 ± 4.78	97.39 ± 0.45	77.57 ± 5.66	68.70 ± 4.05

Table S9. Performance of the RF model in the four-class test with the isolated implementation of RF, the implementation of dimensionality reduction / feature engineering, and the introduction of oversampling to the model. Results from 5-fold cross-validation are given as the balanced accuracy of the model in mean (%) ± standard deviation (%) format.

Study 4: Five-class test

		No. of Features	Train	Validation	Test
RF		131	84.11 ± 2.57	61.03 ± 2.98	60.37 ± 2.93
RF + Dimensionality Reduction	SPCA	131 → 70	81.62 ± 2.19	58.63 ± 5.06	59.57 ± 3.21
	NMF	131 → 100	89.16 ± 1.73	61.52 ± 1.79	53.45 ± 3.32
	LDA	131 → 3	80.34 ± 0.75	61.02 ± 3.90	61.25 ± 4.39
RF + Feature Engineering	SPCA	131 → 163	86.72 ± 1.14	59.88 ± 5.10	58.92 ± 1.98
	NMF	131 → 195	88.49 ± 1.28	62.82 ± 2.69	60.44 ± 1.07
	LDA	131 → 134	**88.54 ± 1.21**	**62.58 ± 4.28**	**64.01 ± 2.25**

			Equaling all classes to the majority class			Oversampling all classes by 50% relatively to the majority class			Oversampling all classes by 100% relatively to the majority class		
			Train	Validation	Test	Train	Validation	Test	Train	Validation	Test
RF + Dimensionality Reduction + Oversampling	Random Oversampler	3	87.43 ± 0.91	61.24 ± 2.70	56.17 ± 5.93	88.45 ± 0.55	62.02 ± 2.85	57.84 ± 4.04	89.85 ± 0.43	61.93 ± 4.72	56.41 ± 5.57
	SVM SMOTE		90.53 ± 1.23	63.78 ± 2.00	58.04 ± 3.36	91.39 ± 0.85	63.21 ± 1.94	60.38 ± 3.12	92.27 ± 0.57	62.96 ± 3.93	60.18 ± 3.65
RF + Feature Engineering + Oversampling	Random Oversampler	134	94.62 ± 0.59	64.19 ± 1.39	63.79 ± 4.55	**96.78 ± 0.48**	**65.41 ± 4.65**	**67.31 ± 3.93**	99.38 ± 0.13	65.42 ± 2.76	61.83 ± 4.78
	SVM SMOTE		94.02 ± 0.43	66.36 ± 3.19	63.38 ± 3.29	96.57 ± 0.56	66.72 ± 2.95	63.14 ± 2.88	97.71 ± 0.49	67.71 ± 4.53	65.61 ± 1.72

Table S10. Performance of the RF model in the five-class test with the isolated implementation of RF, the implementation of dimensionality reduction / feature engineering, and the introduction of oversampling to the model. Results from 5-fold cross-validation are given as the balanced accuracy of the model in mean (%) ± standard deviation (%) format.
